# SLE and Non-Hodgkin's Lymphoma: A Case Series and Review of the Literature

**DOI:** 10.1155/2017/1658473

**Published:** 2017-03-27

**Authors:** Prajwal Boddu, Abdul S. Mohammed, Chandrahasa Annem, Winston Sequeira

**Affiliations:** ^1^Department of Internal Medicine, Advocate Illinois Masonic Medical Center, 836 W. Wellington Avenue, Chicago, IL 60657, USA; ^2^Department of Rheumatology, Rush University Medical Center, 1725 West Harrison Street, Chicago, IL 60612, USA

## Abstract

Systemic lupus erythematosus (SLE) is a multisystem autoimmune disorder punctuated by varied multiorgan complications all along the course of its natural history. Lymphoma represents a relatively well-recognized malignant phenomenon associated with lupus. The cause and effect relationships of lymphoma in SLE have been subject to extensive scrutiny with several studies reporting on clinic-pathologic characteristics and risk factors predicting lymphoma development in SLE. However, the pathogenic role of immunosuppressives in SLE-related lymphoma still remains unclear, and indices to help guide diagnosis, prognostication, therapy, and posttreatment monitoring are yet to be established. In this review, we describe 3 SLE patients who developed non-Hodgkin's lymphoma at different time points of their disease. Through a careful dissection of the aforementioned cases, we intend to apprise readers of the currently available literature surrounding risk factors, management, and prognosis in SLE-related lymphoma. We will also review and discuss the implications of immunosuppressives in SLE-related lymphoma and the role of mycophenolate mofetil in SLE-related primary CNS lymphoma development.

## 1. Introduction

Systemic lupus erythematosus (SLE) is an autoimmune disease with myriad presentations and multisystem complications arising from both underlying disease activity and therapy-related side effects. SLE's association with lymphoma is a well-established phenomenon. A number of studies have reported a higher incidence of lymphoma in the SLE population compared with healthy cohorts [1–3]. The effects of lymphoma development in SLE and its impact on modifying the disease's natural history are a matter of great interest amongst both clinicians and researchers. This has spawned a proliferation of studies examining predictors of lymphoma development, its effect on SLE prognosis, and optimal treatment strategies in its management. In this article, we describe 3 SLE patients who developed lymphoma at different time points of their disease. Of note, all these patients were diagnosed with SLE based on fulfilling the 1997 American College of Rheumatology (ACR) criteria. We will attempt to understand potential factors that may have contributed to disease development in each of the three patients, rationalize treatment strategies that they had received, and identify clinicopathological indices predicting lymphoma development by reviewing the currently updated literature.

## 2. Case #1

A 36-year-old Asian female presented to our hospital with complaints of one month of progressive back pain and two weeks of left lower extremity numbness, cramps, and weakness. She endorsed a two-week history of bowel and bladder retention. She was diagnosed with SLE, over 10 years ago, and was on hydroxychloroquine with reasonable control of her symptoms until a month prior to hospitalization. Her past medical history was also significant for intermittent autoimmune neutropenia. Physical exam at initial evaluation was remarkable for 4/5 strength in both lower extremities with an associated left foot drop. Labs including blood counts and routine serum chemistries were unremarkable at presentation. An MRI of lumbar spine was performed and revealed two peripherally enhancing extradural/subdural masses in the lumbar spinal canal at the L2 and L3-L4 level with additional nodular and linear enhancement of cauda equina nerve roots. A lumbar puncture performed revealed normal CSF cell count, protein 227 mg/dL, and glucose 24 mg/dL; cytopathology was positive for atypical mononuclear cells confirming leptomeningeal involvement. The patient was treated over three days with emergent radiation therapy along with intravenous dexamethasone (10 mg followed by 16 mg daily in divided doses) without further tissue confirmation. Flow cytometry on repeat lumbar puncture, postradiation therapy, showed no diagnostic abnormalities. A L3-vertebral tissue biopsy was performed a day later and showed chronic inflammatory cells and CD20+ necrotic cells consistent with a vanishing lymphoma. Staging with PET and bone marrow biopsy was negative for disseminated lymphomatous involvement. Given the severity of presentation, the patient was treated further with 5 cycles of high dose methotrexate, alternating with rituximab and intrathecal methotrexate. She was started on entecavir, for positive hepatitis B antibody serologies, to prevent possible reactivation of hepatitis while receiving Rituxan. The patient improved clinically over her three-month chemotherapy course after which a restaging PET was performed that showed mild hyper enhancement in the L2 vertebral region. The hyperenhancement on PET was deemed to be postsurgical, based on a preceding core biopsy that was negative for malignancy, and hence an open biopsy was deferred. She completed her localized radiation therapy sessions and proceeded to receive two cycles of consolidation chemotherapy with cytarabine three months later. Six months after her last Rituxan dose, repeat hepatitis B quantitative titers and liver enzymes were negative/normal and entecavir was discontinued. On her most recent follow-up, she continues to make progress and is planned for surveillance monitoring with regular lab work and MRI every 6 months to monitor for disease status and treatment related toxicities.

## 3. Case #2

A 57-year-old African American female presented to the hospital with complaints of headache, fevers, dysarthria, and tremors. She had a 19-year long history of SLE complicated by Class IIIA + Class V lupus nephritis requiring treatment with cyclophosphamide and mycophenolate (MMF). She was also on hydroxychloroquine and had a prolonged course of prednisone (5–10 mg/day). Her other past medical history included anemia of chronic disease, hypertension, hypothyroidism, and listeria meningitis (five months prior to this hospital presentation). Her other relevant medications included trimethoprim sulfa for PJP prophylaxis. Labs at presentation were significant for a Hgb 9 gm/dL, normal WBC and platelet counts, creatinine 2.12 mg/dL, albumin 3.6 mg/dL, bilirubin 0.21 mg/dL, ALP 71 IU/L, ALT 32 IU/L, AST 31 IU/L, LDH 772 IU/L, and uric acid 6.6 mg/dL. Brain MRI was performed for her neurological symptoms and revealed T2-hyperintensive periventricular lesions extending into the corona radiata and internal capsule. A stereotactic brain biopsy was performed which showed large discohesive neoplastic lymphoid cells forming characteristic perivascular cuffing which stained positive for CD20 suggestive of diffuse large B cell lymphoma (DLBCL). Staging PET/CT showed hyper metabolic mediastinal nodes and nodules in the left lower lobe, lesions that were biopsied and showed* Cryptococcus neoformans*. There was no evidence of lymphoma on bone marrow biopsies. A repeat MRI, one month later, demonstrated interval increase in the size of periventricular lesions. A lumbar puncture at this time showed normal cell count with no abnormal cell morphology or flow cytometry. She was started on allopurinol for tumor lysis prophylaxis and fluconazole for recently diagnosed cryptococcosis. She received adjusted high dose intravenous methotrexate for her DLBCL. Her neurological symptoms began to improve after her first session of treatment. At the time of this writing, she is planned to receive further cycles of methotrexate treatment.

## 4. Case #3

A 50-year-old African American male presented to the emergency department with complaints of fever and progressive fatigue over a month. He reported fevers, night sweats, and 17-pound weight loss over this period. His past medical history was significant for SLE with Class II lupus glomerulonephritis and chronic lupus interstitial nephritis for which he was being treated with azathioprine and hydroxychloroquine. Labs at presentation revealed pancytopenia, Hgb 5.6 gm/dL, WBC 3700/mm^3^, and platelets 90000/mm^3^. CT scans of the chest, abdomen, and pelvis demonstrated diffuse lymphadenopathy above and below the diaphragm with no evidence of involvement of extranodal sites. A bone marrow biopsy was performed which showed a hypocellular (30%) marrow with 15–20% of involvement of marrow cells by large CD5, CD19, CD20 positive; Cyclin D1, BCL6 negative B cells with lambda light chain restriction consistent with diffuse large B cell lymphoma. Azathioprine was promptly discontinued. He was determined to have a good prognostic, revised international prognostic index category (R-IPI) Stage IVB DLBCL and treated with 6 cycles of R-CHOP regimen. First chemotherapy cycle was complicated by neutropenic fevers (requiring broad spectrum antibiotics) and progression of kidney disease to end-stage renal disease (ESRD) requiring hemodialysis. Fortunately, he completed the other five cycles without incident. Interim CTs and post-chemo-CT scans with bone marrow biopsy showed complete response with no evidence of residual disease. On repeat evaluation during her follow-up visit, a month after completing treatment, she is asymptomatic with a good recovery of her peripheral blood counts (Hgb 10.2 gm/dL, platelets 195 K/microL, and WBC 3470/mm^3^).

## 5. Summary of the Cases

In summary, patient #1, with a 10-year history of SLE and chronic hepatitis B, presented with primary CNS lymphoma (PCNSL) of leptomeningeal/spinal type and was treated successfully with high dose methotrexate and rituximab. Patient #2 had a 19-year long history of SLE and also presented with a localized CNS lymphoma. Importantly, she was on mycophenolate mofetil (MMF) and cyclophosphamide at the time of her presentation. Mycophenolate therapy was discontinued soon after diagnosis. She was treated with high dose intravenous methotrexate. Patient #3 presented with DLBCL while he was being treated with azathioprine which was later discontinued. He showed good clinical response to chemotherapy and was in complete remission at the end of his R-CHOP regimen. Two of our patients presented with a primary CNS lymphoma, a rare and unusual presentation in SLE patients. Of note, all patients were diagnosed with SLE based on the 1997 ACR criteria.

## 6. Discussion

### 6.1. Incidence and Epidemiology

The association between lymphoma and SLE has been examined in varied detail and studies have reported an increased incidence of lymphoma in SLE [1–3]. Immune dysregulation coupled with the use of immunosuppression may account for the increased incidence of cancer (and lymphoma) in SLE. A recent meta-analysis which included 16 observational studies showed a mildly increased risk of overall cancer in SLE compared with the general population [1]. This risk is most pronounced in SLE-related non-Hodgkin's lymphoma with reported relative risk estimates ranging widely from 4.39 to 44.4 in various studies [1–3].

Based on two studies with a total of 37 patients, SLE patients presenting with lymphoma typically age between 57 and 61 years with the majority of them being women [10, 13]. In a study involving 11 SLE-NHL patients, the mean duration of SLE at the time of NHL diagnosis was found to be 18 years, but cases of NHL occurring prior to concurrently with a diagnosis of SLE have also been reported [5, 7]. Unlike the increased overall cancer risk which is generally confined to Caucasian SLE patients, lymphoma risk in SLE is uniformly higher across all ethnicities [14].

### 6.2. Pathology

The most common histological type of NHL in SLE is the DLBCL type constituting 50–65% of the SLE-lymphoma cases [5, 10, 13]. Gene expression profiling in DLBCL has revealed at least three different subgroups, each driven by different pathophysiological mechanisms and characterized by different responses to therapy. Of these, the “activated B-cell-like DLBCL” type is derived from postgerminal center B cells while the “germinal center B-cell-like DLBCL” type originates from germinal center B cells. DLBCL of the activated B cell type carries a worse prognosis compared with DLBCL of the germinal type (GCB) [10, 15]. Importantly, DLBCL arising in patients with a history of SLE showed an enrichment of non-GCB subtype, and this has potential implications on therapy and prognosis.

### 6.3. Clinical and Laboratory Features

Signs and symptoms like fever and adenopathy are shared clinical features in SLE-related lymphoma and infections complicating SLE. However, a thorough assessment of the duration of lymphadenopathy and chronicity of symptoms, exhaustive exclusion of potential infectious etiologies, and examination of tissue and bone marrow biopsies are essential in arriving at the right diagnosis [16, 17]. Though NHL in SLE is predominantly nodal, extranodal involvement may also occur. Prognosis of NHL in SLE is heavily weighted upon the stage of the disease, with a higher stage predicting for a worsened outcome [18, 19]. Early stage lymphoma can share similar clinical and hematological findings observed in SLE. In addition, lymphadenopathy is very common in SLE, occurring in up to 67% of patients [20]. Hence, the diagnosis often hinges upon a lymph node biopsy or peripheral blood sampling for immunophenotyping to detect a monoclonal cell expansion consistent with lymphoma.

A study examining predictors of increased lymphoma risk in systemic lupus suggested a potential role of cyclophosphamide and high cumulative steroid use [21]. In their sample, disease activity was found to be associated with increased lymphoma risk. A study on Sjogren's syndrome patients with lymphoma identified simple hematological factors such as neutropenia, low complement, and cryoglobulinemia as highly predictive factors in the development of NHL [21]. Of note, we observed our three study patients to have either bicytopenia or pancytopenia for a brief time period just prior to the diagnosis of lymphoma.

### 6.4. Role of Immunosuppressives

Most SLE patients are on potent immunosuppressives for the management of lupus and its complications. Pertinent concerns have been raised against the plausible role of immunosuppressives in lymphomagenesis. The potential association between cyclophosphamide and azathioprine use and lymphoma in SLE has been widely studied in literature. Most of the evidence suggests that immunosuppressives do not increase the incidence of lymphoma [4–12]. Studies, in fact, have shown conflicting results and failed to demonstrate a significant risk of lymphoma with use of cyclophosphamide and azathioprine (see Table 1). The role of other agents like MMF is detailed in the following sections.

### 6.5. Pathophysiology

Biological mechanisms behind lymphomagenesis in SLE remain to be better defined. Constitutive immune activation from prolonged antigen stimulation may result in active lymphocyte proliferation which can eventually terminate into a lymphoma. Bone marrow derived naïve B cells ingress into the lymph node and enter a germinal center reaction, after antigen stimulation, where they proliferate rapidly and undergo class switching and somatic hypermutation [22]. DLBCL subtype is derived from activated B cells suggesting that an increased incidence of lymphoma may be a secondary effect of chronic inflammation in this subgroup [23]. Activating mutations in multi-proto-oncogenes have been reported in DLBCL [24]. It is noteworthy to mention that chromosomal translocation break points in SLE DLBCL have been shown to occur in the very same regions as non-SLE DLBCL [25–27].

Epstein Barr virus (EBV) infection is being increasingly recognized as a potential pathogenetic link to lymphomagenesis. It has been observed that EBV antigens share structural similarities with certain SLE antigens. Additionally, SLE patients tend to have frequent viral reactivations as suggested by higher EBV seroconversion rates [28]. These reactivations are partly the effect of defective EBV-specific T cell suppression allowing enhanced expression of viral genes [28, 29] in SLE patients. It is also interesting to note that EBV infection of B cells upregulates the expression of enzymes like cytidine deaminase expression, an essential mediator in the process of somatic hypermutation [30].

### 6.6. Primary CNS Lymphoma

Primary CNS lymphoma (PCNSL) is a regional type of non-Hodgkin lymphoma and is defined by central nervous system involvement without evidence of concomitant systemic disease. The diagnosis of PCNSL carries a worse prognosis compared with its systemic counterpart [31]. Histologically, about 95% of PCNSL is CD20^+^ DLBCL type [32]. Immunodeficiency predisposes to PCNSL development; however, it has also been associated independently with the presence of autoimmune diseases [33]. Almost all cases of PCSNL reported in SLE patients are associated with the use of immunosuppressives [33–38]. As alluded above, patient #1 was treated with endeavor to prevent hepatitis B reactivation, a potential complication while receiving rituximab [39]. Hepatitis B independently associates with increased PCSNL risk and may be due to HBV causing chronic antigenic stimulation [40, 41]. However, our patient was HBsAg negative and anti-HBs Ab positive, an association not likely associated with an increased risk of PCSNL [40]. This patient was only on hydroxychloroquine, a weak immunosuppressant, when she presented with PCSNL. It is worth mentioning that the use of hydroxychloroquine may actually positively influence the cancer risk in SLE patients [42]. An observational prospective study involving 235 SLE patients with a median 10-year follow-up (156 on hydroxychloroquine) determined a decreased cancer incidence while on hydroxychloroquine [adjusted hazard ratio 0.15 (95% CI 0.02 to 0.99)]. Finally, it currently remains unknown if SLE has an immunosuppressive-independent association with primary CNS lymphoma.

PCNSL in SLE is being diagnosed more frequently since the introduction of MMF in the management of lupus nephritis and post-transplant [35–38]. All case studies including ours were treated long term with mycophenolate indicating that prolonged immunosuppression may be one of the prerequisites for PCNSL development [23, 36–38]. All reported patients had at least 5 years of autoimmune disease. All patients were on mycophenolate 1 gram twice daily dosing for 8 months to 46 months prior to diagnosis. Of the five patients, including ours, three were diagnosed with DLBCL and two had polymorphous B cell lymphoproliferative disorder. Three patients went into complete remission after steroid/rituximab regimen; one patient had progressive disease on the same. Our patient was not assessed for CD20 marker status. MMF induces a compromise in T cell immune function thereby likely causing dysregulated B cell proliferation in the setting of chronic antigen stimulation. In all the reports mentioned above, patients had tumors which were positive for EBV, a factor which may well have contributed to chronic antigen stimulation. The EBV status of our patient's tumor was not evaluated. In this context, EBV episomes tend to be absent in immunocompetent PCNSL patients, and other mechanisms of disease induction might be responsible for lymphomagenesis [43]. Finally, PCNSL in SLE without mycophenolate has also been rarely described [34].

All patients were diagnosed with PCNSL within 1–3 months of onset of neurological symptoms. Rapid onset of neurological symptoms in SLE patients on long term immunosuppressives, especially MMF, should raise concern for PCNSL.

### 6.7. Management and Prognosis

Diffuse large B cell lymphoma is highly aggressive but responds well to the traditional CHOP regimen (cyclophosphamide, doxorubicin, vincristine, and prednisone) [44]. Addition of rituximab to CHOP improves response rates and survival outcomes [45]. The optimal regimen for PCSNL is unestablished, with the traditional CHOP regimens generally proving ineffective due to poor penetrance through the blood brain barrier. Hence intrathecal chemotherapy involving high dose methotrexate and rituximab now forms the standard first line regimen in PCSNL [46]. Patient #1 responded well to the combined high dose methotrexate and rituximab regimen with complete response after completion of his chemotherapy.

Course of these patients has not been well studied and may be poor given the relatively higher proportion of DLBCL non-GCB types and higher disease stage at presenting diagnosis. Rossi et al. reported on the outcomes of 5 patients, who on attaining complete remission after treatment with high dose chemotherapy found that 3 of the 4 patients continued to have persistent autoantibody elevation with frequent lupus flares [19]. The potential of stem cell transplantation in serving a potentially curative role not only in the management of DLBCL but also in inducing prolonged serological remissions of autoimmune disease should be explored in future studies [19, 47, 48].

## 7. Conclusion

There has been a wealth of progress in the understanding of SLE-related lymphoma in the past two decades. The availability of simple hematological/laboratory indices predicting lymphoma development can facilitate early identification of disease and warrant further evaluation in future studies. Although immunosuppressives have no clear role in the pathogenesis of SLE-related lymphoma, reports including ours suggest an etiological role of mycophenolate in PCNSL development. The therapeutic role of allogenic stem cell transplant in curing both lymphoma and SLE holds promise and prognostic risk stratification studies are required to identify patients who best benefit from this therapeutic approach.

## Figures and Tables

**Table 1:** Studies on the role of cyclophosphamide and azathioprine on SLE-related lymphomagenesis.

Ref.	Study design	Pt follow-up	Number of patients with lymphoma/hematological malignancies	Drugs studied	Comments	Outcomes
[4]	Prospective observational	68592 patient months	3	Azathioprine & cyclophosphamide	3/914 patients had lymphoma. 1 was on AZT, 1 on CYP, and 1 on both. 348/911 were on AZT, 188/911 on CYP	No association

[5]	Retrospective	2020	11	Azathioprine & CYP	1 on CYP, 0 on AZT	No association

[6]	Retrospective	219	1	Azathioprine & CYP	0 on immunosuppressives	No association

[7]	Retrospective	784 246 cancer cases and 538 controls without cancer	46	Azathioprine & CYP	Cyclophosphamide: 17.5% of 538 cancer free patients, 7.4% of 246 cancer patients; azathioprine: 34.9% of 538 cancer free patients, 25.0% of 246 cancer patients	No association Cyclophosphamide 2.09 (0.69–6.30) Azathioprine 1.19 (0.48–2.92)

[8]	Retrospective	864	8	Azathioprine & CYP	11 of 28 cancer patients on CYP, 12 of 28 cancer patients on AZP	No association

[9]	Retrospective	5036	75	Azathioprine, CYP, MMF, and prednisone	75 lymphoma cases, only 15 had prior exposure to CYP, 19 to AZA, 9 to methotrexate, and 6 to MMF	No association Cyclophosphamide ever used 2.80 (0.87 to 8.98) Azathioprine (AZA) ever used fully adjusted 0.84 (0.32 to 2.25)

[10]	Retrospective	6438	16	Azathioprine, CYP	Cyclophosphamide: 2/16 in patients with NHL, 3/26 in patients without NHL Azathioprine: 5/16 in patients with NHL, 9/26 in patients without NHL	No association CYP (RR 0.3–3.3) AZP RR 0.9 [0.4–2.1]

[11]	Prospective	5976 patient years	1	CYP	1/49 on CYP developed NHL	No association

[12]	Prospective	17376 patient years	3	AZT, CYP	None on CYP or AZT	No association
